# Therapeutic Time Window of Disease‐Modifying Therapy for Early Alzheimer's Disease

**DOI:** 10.1002/alz70860_097785

**Published:** 2025-12-23

**Authors:** Saki Nakashima, Kenichiro Sato, Yoshiki Niimi, Tatsushi Toda, Takeshi Iwatsubo

**Affiliations:** ^1^ Department of Neurology, Graduate School of Medicine, The University of Tokyo, Tokyo, Japan; ^2^ Department of Neuropathology, Graduate School of Medicine, The University of Tokyo, Tokyo, Japan; ^3^ Unit for Early and Exploratory Development, The University of Tokyo, Tokyo, Japan; ^4^ Unit for Early and Exploratory Development, The University of Tokyo, Bunkyo‐ku, Tokyo, Japan; ^5^ Department of Healthcare Economics and Health Policy, Graduate School of Medicine, The University of Tokyo, Tokyo, Japan; ^6^ Department of Neuropathology, Graduate School of Medicine, The University of Tokyo, Bunkyo‐ku, Tokyo, Japan

## Abstract

**Background:**

Lecanemab and donanemab are disease‐modifying therapies (DMTs) indicated for early Alzheimer's disease (AD), requiring evidence of amyloid positivity, a Clinical Dementia Rating‐Global Score (CDR‐GS) of 0.5 or 1, and Mini‐Mental State Examination (MMSE) scores of 22–30 (lecanemab) or 20–28 (donanemab). Because AD progresses gradually, these eligibility thresholds define a limited “therapeutic time window.” Understanding how long patients remain eligible is critical to optimizing clinical workflows and preventing missed opportunities for treatment.

**Methods:**

We analyzed two longitudinal cohorts: the National Alzheimer's Coordinating Center (NACC) and the Alzheimer's Disease Neuroimaging Initiative (ADNI). 977 NACC participants qualified for lecanemab eligibility and 989 for donanemab, while 719 ADNI participants qualified for lecanemab and 594 for donanemab. Eligibility began once participants first met the specified CDR‐GS and MMSE requirements with confirmed amyloid positivity. Cox proportional hazards models examined baseline variables, including MMSE, CDR‐GS, age, and tau biomarkers, to estimate the time until ineligibility.

**Results:**

Higher baseline CDR‐GS (1 vs. 0.5) and lower baseline MMSE scores significantly predicted a shorter eligibility period across both datasets. The pooled hazard ratio (HR) for CDR‐GS of 1 compared with 0.5 was 1.99 (95%CI: 1.64–2.43), indicating nearly twice the risk of early ineligibility. For each 1‐point increase in baseline MMSE, the HR was 0.70 (95%CI: 0.62–0.80). Median survival estimates showed participants with CDR‐GS of 1 often lost eligibility by 12 months, whereas many with CDR‐GS of 0.5 and higher MMSE remained eligible for up to 24 months.

**Conclusions:**

Baseline CDR‐GS and MMSE emerge as strong predictors of how long individuals remain within the therapeutic time window for early AD DMTs. These findings can guide clinicians in prioritizing patients most at risk of losing eligibility and inform scheduling decisions when faced with extended wait times, ultimately helping to optimize the use of limited healthcare resources.

